# Brain stimulation for chronic pain management: a narrative review of analgesic mechanisms and clinical evidence

**DOI:** 10.1007/s10143-023-02032-1

**Published:** 2023-05-29

**Authors:** Michał Szymoniuk, Jia-Hsuan Chin, Łukasz Domagalski, Mateusz Biszewski, Katarzyna Jóźwik, Piotr Kamieniak

**Affiliations:** 1 Student Scientific Association at the Department of Neurosurgery, Medical University of Lublin, Lublin, Poland; 2 Department of Neurosurgery, Medical University of Lublin, Lublin, Poland

**Keywords:** Chronic pain, Treatment, Deep brain stimulation, Motor cortex stimulation, Non-invasive brain stimulation

## Abstract

Chronic pain constitutes one of the most common chronic complaints that people experience. According to the International Association for the Study of Pain, chronic pain is defined as pain that persists or recurs longer than 3 months. Chronic pain has a significant impact on individuals’ well-being and psychosocial health and the economy of healthcare systems as well. Despite the availability of numerous therapeutic modalities, treatment of chronic pain can be challenging. Only about 30% of individuals with non-cancer chronic pain achieve improvement from standard pharmacological treatment. Therefore, numerous therapeutic approaches were proposed as a potential treatment for chronic pain including non-opioid pharmacological agents, nerve blocks, acupuncture, cannabidiol, stem cells, exosomes, and neurostimulation techniques. Although some neurostimulation methods such as spinal cord stimulation were successfully introduced into clinical practice as a therapy for chronic pain, the current evidence for brain stimulation efficacy in the treatment of chronic pain remains unclear. Hence, this narrative literature review aimed to give an up-to-date overview of brain stimulation methods, including deep brain stimulation, motor cortex stimulation, transcranial direct current stimulation, repetitive transcranial magnetic stimulation, cranial electrotherapy stimulation, and reduced impedance non-invasive cortical electrostimulation as a potential treatment for chronic pain.

**Supplementary Information:**

The online version contains supplementary material available at 10.1007/s10143-023-02032-1.

## Introduction

Chronic pain constitutes one of the most common chronic complaints that people experience, with the prevalence rate ranging between 11% and 40% in the USA [1]. A recent epidemiological study demonstrated that more than 1 in 5 Americans suffer from chronic pain [2], whereas in Europe chronic pain affects about 19% of the adult population with the highest prevalence in Poland and Norway (27% and 30% respectively) [3].

According to the International Association for the Study of Pain (IASP), chronic pain is defined as pain that persists or recurs longer than three months [4]. Patients suffering from chronic pain may experience a drop in their quality of life due to social dysfunctions, sleep disorders, and depression [5, 6]. Moreover, chronic pain has a significant impact not on individuals’ well-being only but also on the economy of healthcare systems. In the USA, estimated economic costs of chronic pain range between $560 and $635 billion annually, including direct healthcare costs and lost productivity [7].

A recently published classification of chronic pain designed by the IASP for the 11th Edition of the International Classification of Diseases (ICD-11) divides chronic pain into chronic primary pain and chronic secondary pain [8]. While the primary pain is related to remarkable emotional distress and/or functional dysfunction without other known causes [8], the secondary pain is a result of an underlying condition such as tumors, injury, surgery, musculoskeletal disease, or nerve damage [9–15].

Despite the availability of numerous therapeutic modalities, the treatment of chronic pain can be challenging. Conventional management of this condition includes oral analgesics as the first-line treatment administered according to the World Health Organization analgesic ladder [16]. However, for cancer-related chronic pain, this low-cost and simple therapeutic tool is effective in 75–90% of patients, only about 30% of individuals with non-cancer chronic pain achieve improvement from opioid treatments [17, 18]. Considering the possible side effects of long-term opioid use (78% overall adverse event rate, including 7.5% serious adverse events) and the risk of addiction, the harms outweigh the benefits of opioid therapy [19]. For this reason, in treatment-resistant cases, dose reduction or discontinuation of opioid treatment may be considered towards alternative therapies [20, 21]. Numerous therapeutic approaches were proposed as a potential treatment for chronic pain including non-opioid pharmacological agents, nerve blocks, acupuncture, cannabidiol, stem cells, exosomes, and neurostimulation techniques [22–27]. Moreover, the role of the psychosocial aspect in chronic pain treatment and the multimodality of therapy is emphasized by recent reports [28–31].

Even though the idea of neurostimulation originated more than a century ago, this therapeutic modality grew the attention of researchers only in the last decades [32]. Numerous studies demonstrated neurostimulation techniques as a potential treatment for a variety of neurological disorders such as epilepsy, Parkinson’s disease, dystonia, and many others [33–36]. Among developed neurostimulation methods, three major groups can be distinguished—brain stimulation, spinal cord stimulation (SCS), and peripheral nerve stimulation [37–40]. Although the clinical use of SCS was recently approved by the Food and Drug Administration as a therapy for chronic pain [41, 42], the current evidence for brain stimulation efficacy in the treatment of chronic pain remains unclear.

Hence, this narrative literature review aimed to give an up-to-date overview of brain stimulation methods, including deep brain stimulation (DBS), motor cortex stimulation (MCS), transcranial direct current stimulation (tDCS), repetitive transcranial magnetic stimulation (rTMS), cranial electrotherapy stimulation (CES), and reduced impedance non-invasive cortical electrostimulation (RINCE), as regards their mechanism of action, clinical efficacy, and common adverse effects, in the treatment of chronic pain.

## Materials and methods

**Fig. 1:**
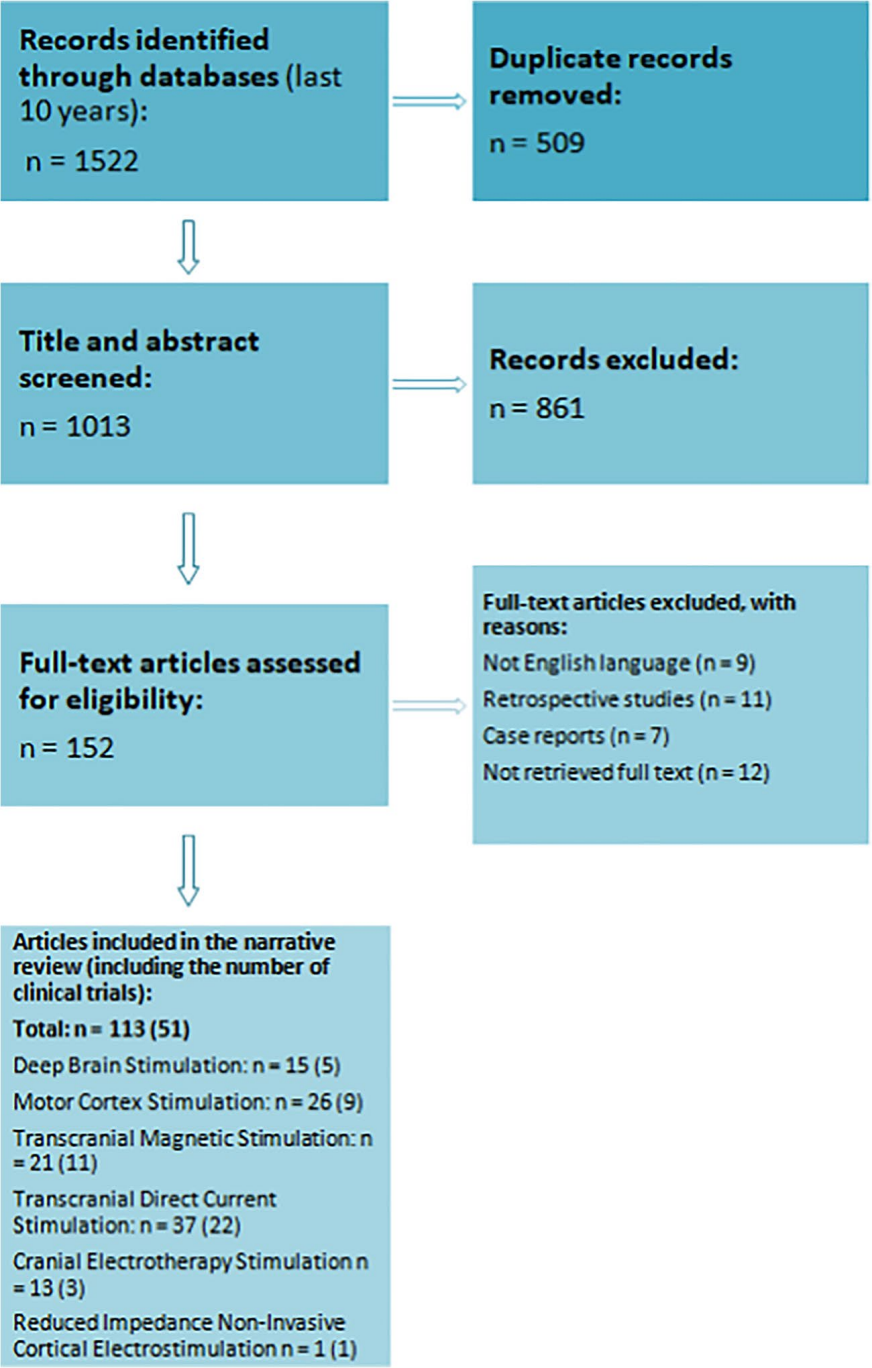
Flow diagram of the literature search strategy

This narrative review was conducted according to the Scale for the Assessment of Narrative Review Articles (SANRA) criteria [43]. A literature search was performed in November and December 2022 based on the MEDLINE (PubMed) database with the use of the following terms: “neurostimulation,” “brain stimulation,” “chronic pain,” “pain,” “deep brain stimulation,” “motor cortex stimulation,” “transcranial direct current stimulation,” “repetitive transcranial magnetic stimulation,” “cranial electrotherapy stimulation,” “reduced impedance non-invasive cortical electrostimulation” (Fig. 1)

The literature search was limited to articles published in the last 10 years (between January 2013 and December 2022) to provide current data and trends in therapeutic outcomes of brain stimulation. Moreover, a comprehensive search of selected papers’ references was conducted to identify further articles of interest. Special attention was devoted to clinical trials. Case reports, retrospective studies, and articles written in languages other than English were excluded.

## Brain stimulation methods for chronic pain

**Fig. 2:**
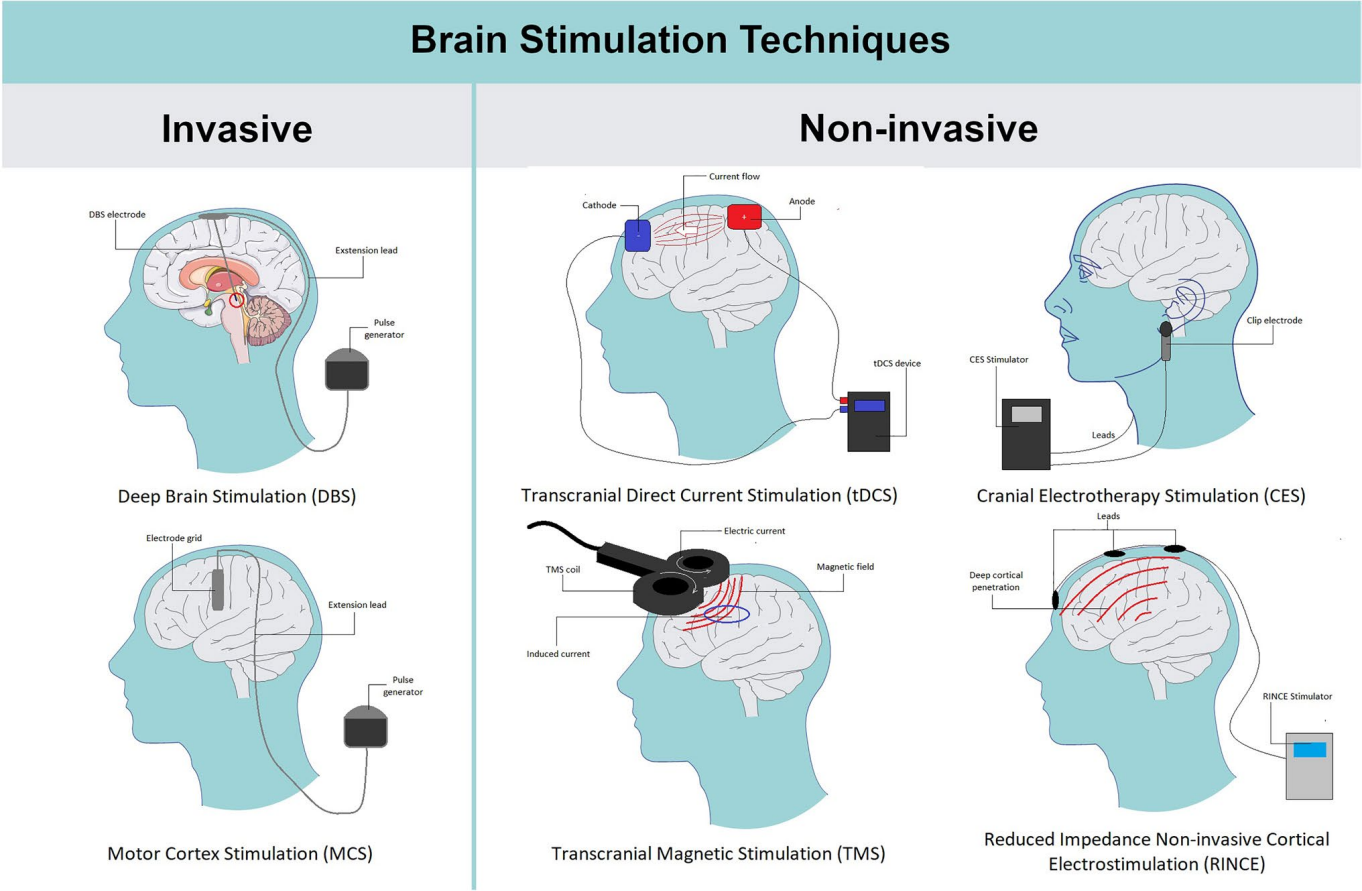
Graphical presentation of available brain stimulation techniques. Parts of the figure were drawn using pictures from Servier Medical Art. Servier Medical Art by Servier is licensed under a Creative Commons Attribution 3.0 Unported License (https://creativecommons.org/licenses/by/3.0/)

Among discussed techniques of brain stimulation, invasive as well as non-invasive methods can be distinguished. Non-invasive techniques are mainly based on the percutaneous stimulation system and include techniques such as tDCS, rTMS, CES, and RINCE. On the other hand, invasive methods require neurosurgical procedures for electrode implantation and include methods such as DBS and MCS (Fig. 2).

### Invasive brain stimulation

#### Deep brain stimulation

DBS is a method of delivering an electric current to the brain through implanted electrodes. It uses high stimulation frequencies that functionally deactivate the neurons present near the electrodes but the fiber pathways can still be stimulated [44].

The most common DBS targets in chronic pain treatment include the periventricular and periaqueductal gray matter (PVG/PAG) and the rostral anterior cingulate cortex (ACC). The choice of simulation target depends on the cause of the present pain. For example, the best approach for stimulation for phantom limb pain is thalamic DBS, which was supported by results of pain reduction ranging from 50.6 to 76.4% in patients participating in the study conducted by Abreu et al. [45]

Drug-refractory chronic cluster headache is among the clinical conditions where the application of DBS may be efficient. The usage of high-frequency discharges has been proven to reduce the occurrence of complaints in 60% of patients, with up to 30% of patients having their pain completely relieved [46]. The location that turns out to be the pulse generator in this type of discomfort appears to be the area between the hypothalamus and the mesencephalon. An alternative technique to the mentioned above is invasive sphenopalatine ganglion stimulation. The method of action involves parasympathetic inhibition using high-frequency stimulation [47]. The success of this method was confirmed by a randomized multicenter study, according to which pain reduction was achieved in 67% of patients with cluster headaches and complete absence of pain occurred in 34% of treated individuals [48]. The results after 24 months showed a pain reduction of 65%, and more than 50% decrease in the frequency of attacks was achieved in 43% of patients. This suggests that using this type of stimulation may serve as a preventive treatment for chronic cluster headaches [47].​

What seems noteworthy is the hypothesis of an endogenous opioid secretion mechanism when the brain is stimulated within the PAG. A study was conducted on a sample of five patients with the DBS system implemented. The researchers used the opioid radiotracer [11C]-diprenorphine (DPN). This compound belonging to the opioid receptor agonists showed high levels of binding in the thalamus, midbrain, and several cortical regions but low levels of binding in the occipital cortex and the pontine nucleus [49]. The results seem to suggest the secretion of endogenous opioids, which prevented DPN attachment. However, despite these reports, which have not been fully confirmed, the exact mechanism remains to be determined. Nevertheless, the potential for disorienting psychoactive, respiratory depression, and other side effects in chronic pain patients who manage only with opioid drugs may be the impetus for an in-depth discovery of the mechanisms at play here and thus originate novel effective and tolerable treatment paradigms to lessen the suffering of patients [50].

Research into the effects of DBS has resulted in the identification of a new anatomical target that appears to be a combination of the sensory and limbic systems, which takes place in a situation of painful sensations. This target turns out to be the posterior insula due to its role as a link between the spinothalamic pathway and the ventroposteromedial nucleus of the thalamus [51]. Also confirming the need to consider this point seems to be the fact that functional imaging has demonstrated activation of the posterior insula on individuals to a level dependent on pain intensity, as well as the phenomenon of neutrality to painful stimuli occurring in patients whose brain lesion has involved the insula.

Considering the hypothesis according to chronic pain results from faulty synchronization between brain networks encoding the somatosensory, affective, and cognitive impulses, DBS seems to be an ideal source of a mechanism to interrupt this severe synchrony [52]. The idea of closed-loop stimulation requires a device that reads brain waves of specific frequency bands from three areas of the brain (primary somatosensory cortex, the dorsal anterior cingulate, and the orbitofrontal cortex) and then determines the signals recognized as a baseline based on them. The device should be able to interpret the stronger signals and generate pulses to interrupt the disturbance experienced as pain. An alternative to this solution can be open-loop stimulation, the difference of which is the absence of the presence of sensors, so in this solution, decoupling of neural signals occurs all the time, or patient-triggered stimulation, in which the patient controls the device, but in this case, ideally, the pain signals should be of somatosensory origin [52].

Although the clinical studies demonstrated favorable results of applying the DBS for chronic pain treatment (Table 1), the number of patients treated by this method is declining. Lack of approval for the clinical use of this method in some countries and improvement of the other treatment approaches are some of the many factors responsible for that phenomenon. Despite that, patients with severe, exhausting neuropathic pain with objective pathology refractory to more conservative treatments seem to be the best candidates for this method [44].

**Table 1:** Summary of clinical trials from the last 10 years (2013–2022) investigating deep brain stimulation (DBS) for various chronic pain conditions

Study	Country	Study design	Sample size	Chronic pain condition	Anatomical target area	Main results	Adverse events
Gopalakrishnan 2018 [53]	USA	RCT	10 (later reduced to 7 participants)	CPSP	VC/VS	Significant improvements in outcome measures sensitive to pain affect The key finding of the present study is that DBS restored pain anticipatory phenomena on the affected extremity, allowing the network to again distinguish cues associated with incoming PS from cues associated with incoming NPS.	N/A
Lempka 2017 [54]	USA	RCT	10	CPSP	VS/ALIC	VS/ALIC DBS demonstrated an acceptable safety profile and statistically significant improvements on multiple outcome measures related to the affective sphere of pain. Several participants maintained ≥50% improvements in the MADRS, BDI, and McGill Affective Pain Rating Index at the 2-year follow-up.	N/A
Belasen 2017 [55]	USA	RCT	19 (11 with chronic pain)	Chronic pain associated with Parkinson’s disease	STN	In the patients with chronic pain, low-frequency stimulation significantly reduced heat detection thresholds as compared with thresholds following high-frequency stimulation.	N/A
Son 2014 [56]	Korea	Open-label single-arm study	9	SCI (*n* = 3); CPSP (*n* = 4); syringomyelia (*n* =1); phantom limb pain (*n* = 1)	VC + epidural MCS	8 patients (89%) adequately responded to treatment after 39 months postsurgery; the percentage of pain relief in the chronic DBS group was 37.5%.	Superficial wound infection (*n* = 1)
Marques 2013 [57]	France	RCT	19	Chronic pain associated with Parkinson’s disease	STN	Significant increase of pain and tolerance mechanical thresholds not only after acute STN-DBS but also after acute levodopa administration. Clinical pain alleviation after STN-DBS cannot be considered merely as a consequence of motor complications improvement and could be attributable to a direct central modulation of pain perception, via increased mechanical pain and tolerance thresholds.	N/A

*RCT* randomized controlled trial, *CPSP* central poststroke pain, *VS/ALIC* ventral striatum/anterior limb of the internal capsule, *STN* subthalamic nucleus, *VC/VS* ventral capsule/ventral striatum, *VC* ventralis caudalis nucleus, *MCS* motor cortex stimulation, *N/*A not available, *PS* painful stimulus, *NPS* nonpainful stimulus

DBS presents the risks of open surgical procedures, including severe complications such as hemorrhages and infections. The concern may also be triggered by the possibility of a stimulation-induced seizure [58].

Depending on the location of the electrodes, symptoms such as paresthesias, muscle spasms (stimulation near the internal pouch), and phosphenes (stimulation near the optic nerve) can be expected. Side effects can also occur with using ordinary therapeutic voltages, but then it is necessary to reposition the electrode in a slightly altered location [59].

#### Motor cortex stimulation

MCS is an invasive neurostimulation method proposed as an alternative treatment for chronic neuropathic pain refractory to the standard therapy [60]. First reports regarding the successful clinical use of MCS for chronic pain date from the early 1990s and concern the treatment of chronic thalamic pain [61]. So far, MCS was investigated in numerous clinical studies as a potential treatment for various chronic pain conditions including central post-stroke pain (CPSP) as well as brachial plexus avulsion, trigeminal neuropathic pain, post-surgical pain, pain after spinal cord injury, and more [62].

Implantation of an MCS stimulator involves craniotomy and placement of the electrode in the part of the precentral gyrus or central sulcus corresponding to the painful area. Based on the anatomical spaces where the stimulator leads can be placed, there exist two types of MCS—subdural MCS and epidural MCS [63]. Although studies did not demonstrate significant differences as regards clinical efficacy between these types of MCS [64], placement of the lead subdurally may be more reasonable in case of significant distance between cortex and dura mater [65].

In MCS, pain reduction is obtained by stimulating the region of the motor cortex appropriate for the painful area reported by the patient [62]. However, the exact mechanisms of action through which MCS relieves pain remain not fully understood. A few hypotheses were proposed to explain the analgesic effects of MCS. One of them concerns the modulation of analgesic pathways in the central nervous system by MCS [66]. Preclinical research initially demonstrated that MCS indirectly activates descending inhibitory pathway through a decrease of thalamic activity and in consequence activation of midbrain periaqueductal gray neurons [67]. The role of the ACC was also considered significant for the development of chronic pain [68]. A recent study demonstrated that ten daily sessions of MCS on the neuropathic pain animal model decreased mechanical allodynia and induced neuronal changes in ACC [69], whereas inhibition of protein kinase M zeta (PKMζ) hampered the effectiveness of MCS. Thus, activation of PKMζ in the ACC may be beneficial to the analgesic properties of MCS. The release of endogenous opioids induced by MCS was suggested as another factor responsible for its analgesic effect [70]. Moreover, it has been proposed that MCS modulates the descending analgesic pathways through the cannabinoid and opioid systems [71]. Therefore, compromising of spinal CB2 cannabinoid receptor activation in some groups of patients may be the cause of opioid as well as MCS resistance. Poor understanding of the mechanism underlying MCS-induced analgesia may be one of the causes contributing to refractory to this therapy in two third of patients with chronic pain [72]. Hence, research in this field should be continued to increase the clinical efficacy of MCS. Clinical trials conducted to date showed inconsistent results (Table 2). Moreover, most of them recruited a limited number of patients and were performed with low-quality methodology. Only four randomized controlled trials (RCTs) have been registered in the years between 2013 and 2022 [73, 78, 81, 85]. Some recent studies demonstrated significant chronic pain reduction in the majority of patients at long-term follow-up [56–73] [78, 81, 85, 79, 83]. In other trials, about 30–40% of patients experienced successful outcomes of chronic pain treatment by MCS at several-year follow-up [75]. However, some research showed unsuccessful outcomes or even stopped before complete data collection due to high complication rates and poor treatment results [81]. In a recent RCT, 39% of patients successfully responded to MCS [73]. However, adverse events were common in this study and concerned the majority of patients. Another RCT demonstrated the long-term benefits of MCS in half of the patients with follow-up ranging from 2 to 9 years [85]. Low rates of clinical efficacy and inconsistent results of available studies may suggest that MCS is beneficial for only specific subgroups of patients with chronic pain. Indeed, some reports suggested that MCS is more effective in the treatment of chronic pain associated with phantom limb pain, facial pain, and complex regional pain syndrome (CRPS) than in the treatment of CPSP or chronic pain resulting from brachial plexus avulsion [44, 73, 88]. On the other hand, in some of the recent studies, the use of MCS achieved significant improvements in patients with chronic pain caused by CPSP and trigeminal neuropathic pain [64, 79, 82, 89]. However, many other neurostimulation approaches have been studied in the treatment of facial pain [90]. One of them—stimulation of the Gasserian Ganglion—resulted in successful pain reduction in 44% of patients with refractory trigeminal neuropathy at 24-month follow-up [91]. Another neuromodulation technique—peripheral nerve field stimulation—provided satisfactory pain relief in 4 of 8 patients with trigeminal neuralgia associated with multiple sclerosis after 24 months of follow-up [92]. Despite their low evidence, these findings decrease the value of MCS in the treatment of facial pain syndromes considering its higher invasiveness. However, studies directly comparing the safety and performance of these methods with MCS in the treatment of facial pain have not been conducted to date.Considering other factors which may influence MCS effectiveness, some authors indicated that visual analogue scale at 1-month post-implantation and successful preoperative rTMS may be potential predictive factors of MCS [85, 89, 76]. Moreover, according to a recent systematic review, the younger age of patients with facial pain was positively related to the definitive MCS implantation rate [93]. Nevertheless, due to the lack of high-quality evidence, further studies should explore predictive factors for successful MCS to establish appropriate indications for this treatment method. As an invasive method, MCS is burdened with a high risk of complications. According to a recent systematic review, the most common adverse events include temporary partial seizures (18%) and wound infections (12%) [89]. In recent studies, epidural hematomas and dural scars have been also observed [79, 87]. Moreover, device failures such as electrode shifts or generator malfunctioning were present in a majority of studies from the last ten years reporting adverse events.

**Table 2:** Summary of clinical trials from the last 10 years (2013–2022) investigating motor cortex stimulation (MCS) for various chronic pain conditions

Study	Country	Study design	MCS type	Sample size	Chronic pain condition	Main results	Adverse events
Hamani 2021 [73]	Brazil	RCT	Epidural MCS	18	Trigeminal neuropathic pain (*n* = 3); CPSP (*n* = 4); brachial plexus avulsion (*n* = 6); phantom limb pain (*n* = 3); CRPS (*n* = 2)	39% of patients adequately responded to treatment in 1-year follow-up with a minimum 2-point or 30% reduction in NRS scores.	Infection (*n* = 1), pseudo-seizures (*n* = 1), intraoperative seizure (*n* = 1), device failure (*n* = 1), discomfort neck (*n* = 6), incision hyperemia (*n* = 3)
Sokal 2019 [74]	Poland	Open-label single-arm study	Epidural MCS	6	Thalamic pain (*n* = 3), trigeminal neuralgia (*n* = 3)	In 5 patients, burst MCS was more effective than tonic mode.	N/A
Henssen 2018 [75]	Netherlands	Open-label single-arm study	Epidural MCS	18	CPSP (*n* = 7); trigeminal neuralgia (*n* = 3); trigeminal neuropathic pain (*n* = 2); idiopathic facial pain (*n* = 2); post-surgical pain (*n* = 1); brachial plexus avulsion (*n* = 1); phantom limb pain (*n* = 1)	Statistically significant improvement in VAS in 3-year follow-up; successful treatment in 38.9% of patients; benefits only in patients with CNS lesion.	Infection (*n* = 3); intraoperative seizures (*n* = 1); device failure (*n* = 1)
Tanei 2018 [76]	Japan	Retrospective study	Epidural MCS (vs SCS)	*SCS group:* 35; *MCS group:* 15	*MSC group:* CPSP (*n* = 9); trigeminal neuropathic pain (*n* =2); SCI (*n* = 2); multiple sclerosis (*n* = 1); brachial plexus avulsion (*n* = 1); *SCS group:* CPSP (*n* = 17); FBSS (*n* = 7); SCI (*n* = 4); peripheral neuropathy (n = 3); CRPS (*n* = 1); other causes (*n* = 3)	53.3% of patients demonstrated a beneficial effect of MCS 12-month post surgery; VAS at 1-month post-surgery may be a predictive factor of the long-term effects (both MCS and SCS).	N/A
Zhang 2018 [77]	China	Retrospective study	Epidural MCS, subdural MCS	16	CPSP	Statistically significant mean VAS and NPSI reduction after a 5.3-year mean follow-up.	N/A
Ivanishvili 2017 [78]	Canada	RCT	N/A	6	CPSP (*n* = 3); atypical facial pain (*n* = 3)	4 patients adequately responded to treatment; cyclization of MCS was not inferior to constant MCS with regards to pain tolerability; the subjects preferred cyclized MCS in program 15min ON/15min.	N/A
Zhang 2017 [64]	China	Retrospective study	Epidural MCS, subdural MCS	16	CPSP	Significant pain reduction (mean follow-up: 28 months); a significant association between preoperative rTMS and effective outcomes was demonstrated.	Intraoperative seizures (*n* = 2), subdural effusion (*n* = 1), electrode shift (*n* = 1)
Rasche 2016 [79]	Germany	Open-label single-arm study	Epidural MCS	36	Trigeminal neuropathic pain	26 patients demonstrated statistically significant pain reduction in VAS 5.6 years after MCS.	Wound infections (*n* = 4), device failure (*n* = 2), seizures and epidural scar (*n* = 1)
Kolodziej 2016 [80]	Germany	Retrospective study	Epidural MCS	20	Central pain (*n* = 8), deafferentation pain (*n* = 3), and neuropathic trigeminal pain (*n* = 9)	95% of patients demonstrated at least satisfactory pain control (at least 60% pain relief).	Device failure (*n* = 3), wound infection (*n* = 1), epidural hematoma (*n* = 1)
Radic 2015 [81]	Canada	RCT	Epidural MCS	12	Brachial plexus avulsion (*n* = 6); phantom limb pain (*n* = 2); CRPS (*n* = 3); deafferentation pain (*n* = 1)	Trial suspended due to adverse effects, lack of significant change in VAS, no significant changes in other measured outcomes.	Device failure (*n* = 1); infection (*n* = 1); focal motor seizures (*n* = 2); anxiety following seizure (*n* = 1); panic attacks (*n* = 1)
Sokal 2015 [82]	Poland	Retrospective study	Epidural MCS	14	CPSP (*n* = 7), atypical facial pain (*n* = 2), brachial plexus avulsion (*n* = 3), phantom pain, (*n* = 1), pain in syringomyelia (*n* = 1)	Over 80% pain reduction in 31% of the patients in the long term; 50–80% pain reduction in 23% of the patients in the long term; the highest efficacy in post-stroke or post-hemorrhagic thalamic pain.	Transient seizures (*n* = 3); wound infection (*n* = 1); device failure (*n* = 1)
Isagulyan 2015 [83]	Russia	Open-label single-arm study	Epidural MCS + pre-op rTMS	20	CPSP (*n* = 4), atypical facial pain (*n* = 4), phantom limb pain (*n* = 3), brachial plexus injury (*n* = 4), spinal cord injury (*n* = 3), CRPS (*n* = 1), multiple sclerosis (*n* = 1)	14 patients adequately responded to MCS with a reduction in the pain intensity ranging from 25 to 60% after a mean follow-up of 49.3 months; the effect of pre-op rTMS was not statistically significant.	Device failure (*n* = 1); infection (*n* = 2)
Im 2015 [84]	Korea	Retrospective study	Epidural MCS	21	CPSP (*n* = 10), central pain after SCI (*n* = 6); peripheral neuropathic pain (*n* = 5)	76.2% of patients achieved treatment success after 53-month follow-up; only the patients with poststroke pain and peripheral neuropathic pain achieved significant pain reduction (>30% pain relief).	N/A
Andre-Obadia 2014 [85]	France	RCT	Epidural MCS + pre-op rTMS	20	CPSP (*n* = 11); trigeminal neuropathy (*n* = 4); cervical spinal pain (*n* =2); brachial plexus avulsion (*n* = 2); ulnar nerve injury (*n* = 1)	10 patients achieved long-term (6-year follow-up) benefits in pain reduction.	N/A
Sachs 2014 [86]	Canada	Retrospective study	Epidural MCS	14	Trigeminal neuropathic pain (*n* = 7); phantom limb pain (*n* = 3); CPSP (*n* = 1); spinal cord injury (*n* = 1); medullary cavernous malformation (*n* = 1); facial hemangiopericytoma (*n* = 1)	Only 2 patients experienced >50% pain reduction (mean follow-up: 55.5 weeks).	Infection (*n* = 2); intraoperative seizures (*n* = 3)
Buchanan 2014 [65]	USA	Retrospective study	Epidural MCS, subdural MCS	8	CPSP (*n* = 2); facial pain (*n* = 5); phantom limb pain (*n* = 1)	Statistically significant decrease of mean VAS score at 3-month follow-up.	Transient seizures (*n* = 1)
Delavallee 2014 [87]	Belgium	Open-label single-arm study	Subdural MCS	18	Trigeminal neuropathic pain (*n* = 7); CPSP (*n* = 3); brachial plexus avulsion (*n* = 2); C2 avulsion (*n* = 2); SCI (*n* = 1); cubital nerve injury (*n* = 1); CRPS (*n* = 1); phantom limb pain (*n* = 1)	77.7% of the patients achieved >50% pain relief at 3-year follow-up.	Transient seizures (*n* = 4); superficial wound infections (*n* = 4); subdural scar (*n* = 1)

*CPSP* central post-stroke pain, *CRPS* complex regional pain syndrome, *FBSS* failed back surgery syndrome, *N/A* not available, *NPSI* neuropathic pain symptom inventory, *NRS* numerical pain rating scale, *RCT* randomized controlled trial, *SCI* spinal cord injury, *VAS* visual analogue scale, *Vc* ventralis caudalis

### Non-invasive brain stimulation

#### Transcranial direct current stimulation

tDCS is a pioneering non-invasive method, constantly being improved by current research. The application of this method is diverse. Apart from chronic pain described in this work [94], tDCS has already been used in patients with various neuropsychiatric conditions such as depression, mania, schizophrenia, obsessive-compulsive disorder, panic, and post-traumatic stress disorder [95–98]. In addition, recent studies have shown that tDCS can also achieve positive results in Alzheimer’s disease treatment [99]. Additionally, tDCS can be used combined with techniques such as transcranial magnetic stimulation (TMS), fMRI, and electroencephalogram (EEG) to study how stimulation modulates cortical excitability [100]. It has proven to be safe, portable, and cost-effective [101].

Regarding the tDCS mechanism of action, it involves modulating the activity of the brain which is conditioned by the supply of a small amplitude (usually no more than 2 mA) for a short period (from 10 to 30 min). Typically, the power source used for this test is a battery with two electrodes: an active (polarizing) and a reference electrode. They work according to basic neurophysiology. If the active electrode is an anode, it stimulates the motor cortex to become more excitable. If the cathode is the active electrode, the excitability of the motor cortex is reduced. It has been observed that when anode current is used, connection to the cortical region under the target electrode is easier. On the other hand, when cathode current is used, connection to the cortical region of the brain is inhibited. This is because the placement of the electrodes is as follows: at least one of the electrodes is placed on the scalp—it is through it that electronic currents, causing the polarization of neuronal cell membranes, pass through the skull to reach the brain, thanks to which the level of stimulation of the cortex increased or decreased [94]. The 10–20 EEG system can be applied to determine the location of the electrodes. Most often, the arrangement of the electrodes is in the supraorbital area. However, some studies showed that different localizations of the electrodes may affect the quantity and quality of the current delivered to the brain, which results in different intensities of stimulation delivered to the brain [102].

It should be emphasized that the size and shape of the placed electrodes are essential for successful stimulation. The density of the flowing current depends on this, which determines the total stimulation dose that can be administered to the subject. So far, according to current literature, the safe dose value cannot exceed 216 C/cm^2^ [103]. The most commonly used electrodes range in size from 25 to 35 cm^2^ (5 × 5 cm and 5 × 7 cm) [94]. When it comes to allowing the current to pass through the cerebral cortex, saline is usually used for this. It is recommended to use small saline containers (e.g., 20-ml bottles) that allow a little control of the amount of liquid applied to the sponges [94]. Alternatively, an electroconductive gel (such as EEG paste) can be used. It is applied to the base of the rubber electrode, so there is no need for sponge bags as with the use of saline. However, the gel can also dry quickly due to the temperature emitted by the electrode, which increases the risk of scalp burns [94].

The use of tDCS in the treatment of chronic pain has also been repeatedly described in medical literature. It gave the expected effects of reducing chronic pain when used in cases such as knee osteoarthritis [104], treating joint pain as chikungunya virus complication [105], several times in the treatment of fibromyalgia pain [106, 107], and treatment of multiple sclerosis [108]. However, in some studies, tDCS did not produce the expected results. In the study by Luedtke et al., five-day stimulation did not reduce disability associated with non-specific chronic low back pain [109]. O’Neill et al. also failed to prove that tDCS would be effective in patients with neuropathic pain [110]. Research is also underway on the use of tDCS as a method to inhibit neural changes caused by chronic stress. tDCS would then be used before the patient is exposed to chronic stress, which in the future may lower the pain threshold and lead to hyperalgesia [111].

Several RCTs have been registered using tDCS (Table 3). The results varied depending on the electrodes’ position, stimulation duration, and current intensity. Fregni et al. reported pain reduction after 16 days of active tDCS, and in another study, they increased the pain reduction effect to 21 days [128, 129]. Boggio et al. scored up to 28 days of pain relief [130]. Then, Mori et al. received a pain reduction effect lasting up to 28 days [131]. Soler et al. extended the effect of reducing pain up to 12 weeks [132]. As we can see, research on the treatment of pain using tDCS was conducted years ago and already had satisfactory results. Recently, De Souza et al. conducted a study on 58 women in the chronic phase of CHIK disease [105], with an average age of 52.85 years. This group was randomly divided into two: an active group (active-tDCS)—M1-S0—2 mA in a 20-min session was used, and a sham group (sham-tDCS). VAS and Brief Pain Inventory were used to assess pain, and functional capacity was assessed using a Health Assessment Questionnaire. After analyzing the results, six non-consecutive active tDCS sessions on M1 significantly reduced chronic joint pain associated with CHIK. However, no change in functional capacity was noted in any patients. People with epilepsy, metal implants in the stimulation sites, a history of alcohol abuse, breastfeeding women, and pregnant women were excluded from the study. An analysis of the cumulative percentage of respondents [105] showed that 79.31% of active-tDCS participants had a VAS score improvement of more than 30% compared to sham-tDCS. NNT (number needed to treat) calculated in this study was 2, which means two patients had to undergo this technique for one more to have the desired effect. Moreover, anodal tDCS applied for approximately 15–20 min significantly reduced phantom limb pain in amputees [133].

**Table 3:** Summary of clinical trials from the last 10 years (2013–2022) investigating transcranial direct current stimulation (tDCS) for various chronic pain conditions

Study	Country	Study design	Sample size	Chronic pain condition	Main results	Adverse events
De Souza 2021 [105]	Brazil	RCT	58	CHIK	Significant pain reduction was observed in the tDCS group compared to the sham group (*n*=14.303); no significant difference in functional capacity was observed (*n*=2797).	N/A
Ahn 2017 [104]	USA	RCT	40	Knee OA pain	Active tDCS significantly reduced Numeric Rating Scale of pain compared to sham tDCS after completion of the five daily sessions, and remained up to 3 weeks (*n*=20).	N/A
Chang 2017 [112]	Australia	RCT	57	Knee OA pain	All participants in the AT+EX group (*n*=13) and in the ST+EX group (*n*=12) reported an improvement in their knee OA symptoms following treatment.	Headache (*n* = 1); pain during primary stimulation (*n* = 1)
Harvey 2017 [113]	Canada	RCT	14	OA (*n* = 4); CLBP (*n* = 4); spinal cord injury (*n* = 1); other chronic neuropathic pain (*n* = 5)	Active but not sham tDCS significantly reduced pain (*p* >0.05). No change was observed in sleep parameters, in both the active and sham tDCS groups (all *p* ≥0.18).	N/A
Hazime 2017 [114]	Brazil	RCT	92	CLBP	The results suggest that tDCS + PES (mean reduction [MR] = −2.6, CI95% = −4.4 to −0.9) and PES alone are effective in relieving CLBP in the short term. However, only tDCS + PES induced a long-lasting analgesic effect. tDCS alone showed no clinical meaningful pain relief.	N/A
Khedr 2017 [106]	Egypt	RCT	40	Fibromyalgia	The effect of treatment differed in the two groups with higher improvement in the experimental scores of the patients in the real tDCS group (*p* = 0.001 for WPI, SS, VAS, pain threshold, and 0.002, 0.03 for HAM-A, HAM-D respectively). Ten sessions of actual tDCS over an M1 may induce pain relief and mood enhancement in patients with fibromyalgia.	N/A
Lagueux 2018 [115]	Canada	RCT	22	CRPS	GMI+tDCS induced no statistically significant reduction in pain compared with GMI+sham tDCS (*n*=22).	N/A
Thibaut 2017 [116]	USA	RCT	33	Spinal cord injury	Baseline comparisons between stimulation conditions showed no significant a priori differences except for gender (chi-square: 4.90; *p* =0.009).	N/A
Ayache 2016 [108]	France	RCT	16	Multiple sclerosis	Compared to sham, active tDCS yielded significant analgesic effects according to VAS and BPI global scales. The mean VAS_0−100_ pain ratings 7 days before and 7 days after stimulation showed significant decrease after active tDCS (*p* = 0.024), but no change after sham tDCS (*p* = 0.66). Analogously, the mean VAS_0−100_ pain ratings for days 1–3 before and after stimulation showed significant decrease after active tDCS (*p* = 0.021), and no improvement after sham tDCS (*p* = 0.56).	N/A
Brietzke 2015 [117]	Brazil	RCT	28	HCV	tDCS decreased VAS scores (*p* < 0.003), with mean decrease of 56%.	N/A
Mendonca 2016 [118]	Brazil	RCT	45	Fibromyalgia	This study has demonstrated that neuromodulation with tDCS, in association with aerobic exercise training, in fibromyalgia patients effects greater decreases in pain intensity than the individual techniques. The improvement from baseline was: for the tCDS group = 20.8%, for the AE group = 19.1%, for the tCDS/AE group = 31.5%.	Headache (*n* = 3); neck pain (*n* = 1); tingling (*n* = 5); skin redness (*n* = 11); somnolence (*n* = 5); concentration issues (*n* = 1)
Volz 2016 [119]	Germany	RCT	20	Chronic abdominal pain	The analgesic effects observed are unrelated to inflammation and disease activity, which emphasizes central pain mechanisms in CAP (*n*=20).	N/A
Donnell 2015 [120]	USA	RCT	24	Chronic myofascial pain	There were significant improvements for clinical pain and motor measurements in the active HD-tDCS group compared to the placebo group: responders with pain relief above 50% in the VAS at four-week follow-up (*p*=0.04); pain-free mouth opening at one-week follow-up (*p*<0.01); and sectional pain area, intensity and their sum measures contralateral to putative M1 stimulation during the treatment week (*p*<0.01).	N/A
Luedtke 2015 [109]	Germany	RCT	135	CLBP	This results of this trial on the effectiveness of transcranial direct current stimulation for the reduction of pain and disability do not support its clinical use for managing non-specific chronic low back pain (*n*=135).	N/A
Ngernyam 2015 [121]	Thailand	RCT	20	Spinal cord injury	Research revealed a significant decrease in pain intensity from pre- to post-session for active tDCS treatment (0.800, 95% CI = 0.410 to 1.190; *p* < 0.001) but no statistically significant change in pain intensity for the sham condition (0.025, 95% CI = −0.049 to 0.549; *p* = 0.096). The active treatment condition (anodal tDCS over M1) but not sham treatment resulted in significant decreases in pain intensity.	N/A
Sakrajai 2014 [122]	Thailand	RCT	31	Myofascial pain syndrome	After 4 weeks, 15 (94%) of the participants in the tDCS group reported a 50% or greater reduction in average pain intensity, while only 7 (47%) of the sham group participants reported these levels of pain reduction.	N/A
Hagenacker 2014 [123]	Germany	RCT	17	TN	Anodal tDCS over 2 weeks ameliorates intensity of pain in TN. RS (verbal rating scale) decreased after anodal stimulation from baseline by 18% (±SD 29%), while sham stimulation led to an 11% (±30.8%) increase of VRS. Attack frequency was not significantly decreased between sham or anodal stimulation (*p* = 0.123). One patient was completely pain free after anodal stimulation.	N/A
Souto 2014 [124]	USA	RCT	20	Chronic pain HTLV-I infected patients	There were 8 (80%) responders (reduction of 50% or more in pain intensity) in the tDCS group and 3 (30%) in the sham group (*p* =0.03). Both groups demonstrated improvements for most associated factors evaluated. However, there was no difference in between-group comparison analyses.	Mild adverse events were reported by 100% of patients in the tDCS group and 90% in the sham group.
Wrigley 2013 [125]	Australia	RCT	10	Spinal cord injury	In this trial, tDCS did not provide any pain relief in subjects with neuropathic SCI pain (*n*=10). A similar lack of effect was also seen after sham treatment.	N/A
Jensen 2013 [126]	USA	RCT	31	Spinal cord injury	Very weak and mostly non-significant associations were found between changes in EEG-assessed brain activity and pain (*n*=31).	N/A
Kim 2013 [127]	Korea	RCT	60	PDPN	The reduction in VAS (visual analog scale) for pain was sustained after 2 and 4 weeks of follow-up in the M1 group compared with the sham group (*p*<0.001, *p*=0.007). Significant differences were observed among the three groups over time in VAS for pain (*p*<0.001), CGI score (*p*=0.01), and PT (*p*<0.001). No significant difference was observed among the groups in sleep quality, anxiety score, or BDI score immediately after tDCS.	N/A
Villamar 2013 [107]	USA	RCT	18	fibromyalgia	Found that both active stimulation conditions led to significant reduction in overall perceived pain as compared to sham (*n*=18).	N/A

*CHIK* chikungunya disease, *OA* osteoarthritis, *TN* trigeminal neuralgia, *CLBP* chronic low back pain, *CRPS* complex regional pain syndrome, *PDPN* painful diabetic neuropathy, *HTLV-I* human T lymphotropic virus type I, *VAS* visual analog scale

A recently updated version of the original Cochrane systematic review on the effectiveness of the tDCS method in the treatment of chronic pain [134] considered 747 participants (22 studies involved) and showed that pain intensity as measured by a visual analog scale was reduced by 17% with this that the quality of the evidence was assessed as very low, meaning that the results are likely to be significantly different from the estimated effect. A meta-analysis of the tDCS studies compared to the sham quality of life (measured using different scales in included studies) in the short term showed a positive effect (SMD 0.66 95% CI 0.21 to 1.11, low-quality evidence). tDCS may have short-term effects on chronic pain and quality of life, but multiple sources of bias may have contributed to the observed effects.

Although the use of the tDCS method has reported not many adverse events during many years of research, it has been discovered that there may be mild but also temporary side effects, such as headache, itching of the skin in places of stimulation, moderate fatigue, reddening of the skin under the electrode, difficulty concentrating, severe mood swings, and nausea [94]. As we can see, however, the side effects are insignificant compared to the achieved outcomes of therapy. It was even recognized that symptoms such as moderate fatigue could be related to participation in the experiment, and might not necessarily result from the effect of the tDCS method itself on the body [94]. The most common side effect reported by the respondents is skin itching, and although it usually disappears after the current stabilizes, methods have been developed to alleviate this effect [135].

Namely, the application of a moderate saline solution to the storage bag, using the increase/decrease procedure when tDCS is turned on or off, and the use of a smaller size of the electrodes significantly reduce the itching effect of the skin at the stimulation sites. However, these discounts should not be abused because, for example, when using electrodes of small size, the cost of the method may increase due to the need for a change of density and amount of current. While the research was conducted to monitor potential side effects, an adverse reaction questionnaire was published [136]. The questionnaire covered the 10 most common ailments: headache, neck pain, scalp pain, tingling, itching, burning sensation, redness of the skin, drowsiness, problems with concentration, and mood changes. Each item required participants to answer the question “Do you experience any of the following symptoms or side-effects?” on a 4-point scale: 1 = absent, 2 = mild, 3 = moderate, 4 = severe. However, since the publication of this questionnaire, only a few research groups have used it [137].

Moreover, regarding the side effects of tDCS therapy, special attention should be paid to the often overlooked issue. Namely, it is possible to direct the current towards important body areas, including the heart, respiratory system, and autonomic regions of the brainstem. During the initial tDCS experiments, it was noted that one participant experienced a brief episode of respiratory depression during stimulation when the electrode was placed outside the cerebral leg [138]. However, it used a current of 3 mA, a value above the current safety threshold of 2 mA. Therefore, to maximize the safety of this method, it is crucial to follow the correct current values.

#### Repetitive transcranial magnetic stimulation

TMS is a non-invasive treatment used to address certain neurological and psychological disorders (such as Parkinson’s disease, Alzheimer’s disease, and depression), by inducing depolarization of the neurons in the brain [139–141]. This is achieved by transmitting a strong current through a wire from a machine to a circular wire, which initiates a magnetic field in a perpendicular direction based on Faraday’s law of induction. When the circular wire is applied to the scalp, the time-changing magnetic field induces the current in the axons beneath the scalp, running in the opposite direction to the current in the coil, thereby stimulating the brain tissue [142].

There are 3 main types of TMS: single-pulse TMS, paired-pulse TMS, and trains of repetitive stimuli (repetitive TMS, or rTMS). In this article, we will only focus on rTMS. Typically, a single-pulse TMS lasts for only a few seconds. Therefore, to prolong its effects, repetitive TMS is used. rTMS works by firing the single-pulse stimuli repeatedly at a specific frequency, intensity, and time duration, either to inhibit or to stimulate the activity of a specific cortical area that is applied [143]. In rTMS, two different subgroups can be used for treatment based on their purpose. One is high-frequency rTMS (HF-rTMS), with frequencies ≥ 5 Hz, whereas the other is low-frequency rTMS (LF-rTMS), with frequencies ≤ 1 Hz. HF-rTMS can increase cortical excitability, and MEP size (i.e., motor-evoked potential, electric potential in the motor pathway induced by TMS) [144], and provoke intercellular interactions. As a result, it can enhance and facilitate cell proliferation, focal cerebral blood flow, and synaptic plasticity [142].

In studies of patients with neuropathic pain following a stroke, it has been shown that rTMS may help reduce pain by activating inhibitory pathways and stimulating neurogenesis [142]. rTMS has a range of applications, including post-stroke recovery [145], depression (especially major depression and treatment-resistant depression) [146], fibromyalgia [147], and other neuropathic pains [47].

The mechanism of rTMS on living animals is complicated, yet fascinating. This includes its effects on oligodendrocytes, astrocytes, microglia, and stem cells, which can result in the improvement of survival and maturation in oligodendrocyte stem cells, and the enhancement of neuronal metabolic activity and plasticity in astrocytes [142]. Although many studies proved the efficacy of TMS in treating depression and other psychological problems, the evidence supporting the use of rTMS for pain relief is not strong enough. Additionally, the mechanism of rTMS in reducing neuropathic pain remains unclear.

However, astrocytes have been suggested to play an essential role in suppressing pain. They help form the blood-brain barrier, which acts as a border wall protecting the brain from pathogens and allowing only certain amounts of small molecules, ions, and nutrients to move across the wall. Astrocytes also regulate the metabolism of neurons, phagocytose synapses, and remove debris. The inflammatory molecules, including neuronal nitric oxide synthase, glial acidic fibrillary acidic proteins, and 5-bromo-2-deoxyuridine, are released in astrocytes during the inflammatory process [142, 148]. The levels of these inflammatory agents are downregulated with the use of HF-rTMS (20Hz) treatment, which indicates that the technology can suppress the release of inflammatory substances and thus reduce neuropathic pain. In addition to the anti-inflammatory benefits, an increase in the level of anti-inflammatory mediator IL-10 has been observed after HF-rTMS (10 Hz) treatment, leading to greater neuronal plasticity and recovery after neuronal damage [142].

Thalamus is responsible for relaying and processing pain signals before transmitting them to the cerebral cortex. Some studies suggest that HF-rTMS may influence the activity of the cortical thalamic tract and inhibit the spinothalamic pathway in the ascending pathway and thalamic nuclei. The spinothalamic tract can be divided into two parts: the anterior spinothalamic tract, which is responsible for crude touch and pressure sensation, and the lateral spinothalamic tract, which carries sensations of pain and temperature. By inhibiting the ascending tract, HF-rTMS can reduce the transmission of nociceptive signals to the cerebral cortex and thalamus, thereby reducing the sensation of pain [148].

So far, all the research is only involved a limited number of people, which restricted the accountability of using rTMS to treat patients with chronic pain. However, it is still worth examining previous research (Table 4). Several studies have targeted MCS, which has shown greater pain reduction with longer session durations and more frequent stimulation using HF-rTMS [160]. In the results of four studies [149, 157, 158, 156], the levels of pain reduction are shown to be at least 40% of relief (a clinically significant reduction in pain intensity is approximately 30%), which implies the potential clinical value of the treatment in neuropathic pain. Although it appears to be clinically relevant, the small number of volunteers and a small number of sessions (5–10) and a short period of follow-up (<3 weeks) make it unreliable to put the technique into clinical practice. Thus, more patients need to be involved in the research, and longer sessions and durations of follow-up are required.

**Table 4:** Summary of clinical trials from the last 10 years (2013–2022) investigating transcranial magnetic stimulation (TMS) for various chronic pain conditions

Study	Country	Study design	Sample size	Chronic pain condition	Anatomical target area	Main results	Adverse events
Saisanen 2022 [149]	Finland	RCT	20	Chronic facial pain	Primary motor cortex M1	8 patients (40%) experienced significant pain relief. 4 (20%) exhibited a modest effect, 4 (20%) patients had a slight, clinically non-significant benefit, 4 (20%) patients experienced worsening of pain. Female gender, shorter duration of pain and low Beck Anxiety Inventory scores showed a trend towards a better outcome (*p*=0.052, 0.060 and 0.055, respectively).	Tiredness (*n*=3, in 20Hz rTMS) Headache (*n*=1, in 20Hz rTMS) Dizziness (*n*=2) Pain in the head (*n*=6) Nausea (*n*=1) Poor sleep (*n*=1) Ophthalmic branch tickling (*n*=1) Twitching of hand (*n*=1) Tingling in hand (*n*=1) Tightness in the chest/crushing chest pain (*n*=1)
Bursali 2021 [150]	Turkey	RCT	20	FBSS	Primary motor cortex M1	Significant improvements were achieved in DN4, ODI, BDI, and PSQI scores in the r-TMS group in comparison to the sham group. Achieved improvements in the r-TMS group in terms of VAS, DN4, ODI, BDI, and PSQI scores were sustained at the third month.	Mild headache (real group, *n*=1)
Kumar 2021 [151]	India	RCT	20	Chronic migraine	Primary motor cortex M1	A significant reduction in the mean VAS rating, headache frequency, and MIDAS questionnaire in real rTMS group, and remained steady after 1 month of follow-up.	N/A
Mattoo 2019 [152]	India	RCT	30	Chronic tension-type headache	Motor cortex	The NRS (numerical rating scale) score of headache reduced from 5 to 3.5 in placebo group, and from 5 to 1 in rTMS group. A significant pain intensity in rTMS group more than sham group.	N/A
Choi 2018 [153]	South Korea	RCT	12	CPSP	Primary motor cortex M1	The NRS score of the rTMS group was significantly lower than the sham group score during and after HF-rTMS sessions.	N/A
Fitzgibbon 2018 [154]	Australia	RCT	26	Fibromyalgia	Left DLPFC	Active group compared to sham treatment group had significantly greater improvement in the physical fatigue (*p* = 0.045) and general fatigue (*p* = 0.023) scales at the 1 month follow-up. The active group was significantly more likely (2.84 times) to achieve a minimum 30% improvement in pain intensity ratings (*p* = 0.024).	Site discomfort (active, *n*=4; sham, *n*=1) Headaches (active, *n*=4; sham, *n*=3) Neck pain (active, *n*=0; sham, *n*=2) Nausea (active, *n*=1; sham, *n*=2) Dizziness (active, *n*=1; sham, *n*=0) Other (active, *n*=1; sham, *n*=1)
Choi and Chang 2018 [155]	South Korea	RCT	24	Chronic hemiplegic shoulder pain	Primary motor cortex M1	rTMS group showed a significant decrease in the NRS score at 1 day, and 1, 2, and 4 weeks after finishing rTMS sessions, with no significant change in the sham group. The NRS score after the rTMS sessions reduced by 30.1% at 1 day, 29.3% at 1 week, 28.0% at 2 weeks and 25.3% at 4 weeks.	N/A
Nurmikko 2016 [156]	UK	RCT	40	Chronic neuropathic pain	Primary motor cortex M1 (site A and site B)	rTMS-M1 resulted in greater TOTPAR than that of SHAM. Moreover, ≥15% pain relief after rTMS stimulation was observed. Addition of stimulation over site B (a site over the affected M1 defined as the external reorganized cortical area) improved the responder rate by 58% compared with site A (motor hot spot).	Headache (*n*=9) Dizziness (*n*=5) Sleepiness (*n*=13) Transient increase in pain (*n*=11) Nausea (*n*=8) Pins and needles in face or extremities (*n*=8)
Leung 2016 [157]	USA	RCT	24	Mild traumatic brain injury-related headache	Motor cortex	Pain intensity decreased more significant in real group (from 5.7 ± 1.9 to 2.2 ± 2.7), whereas in sham group reduced from 4.6 ± 1.3 to 3.5 ± 2.0. A significantly (*p* = 0.035) higher percentage of the subjects in the REAL group (58.3%) demonstrated at least a 50% headache intensity reduction at posttreatment 1-week assessment compared with the SHAM group (16.6%).	Transient local tenderness (real group, *n*=1) Mild degree of transient dizziness (real group, *n*=1; sham group, *n*=1)
Umezaki 2015 [158]	USA	RCT	20	Burning mouth syndrome	Left DLPFC	In the real group, pain intensity decreased 67%, and 75% of the patients reported >50% pain decrease on final assessment. There was significant pain reduction in subjects in the real group immediately after 1 week of treatment, whereas there was none in those in the sham group.	N/A
Fricova 2013 [159]	Czech Republic	RCT	59	Chronic orofacial pain	The area of motor cortex that corresponds to the chronic orofacial pain	The sham group had an average VAS score of 5.7 throughout the treatment. The real group receiving 10 Hz rTMS had decreased VAS scores from 5.9 to 4.6 in the first week and then increased to 5.3; the real group receiving 20 Hz rTMS had decreased VAS scores from 5.5 to 4.5 in the first week and further decreased to 4.0 by the third week, with no further change.	N/A

*RCT* randomized controlled trial, *FBSS* failed back surgery syndrome, *CPSP* chronic poststroke pain, *DLPFC* left-hemisphere dorsolateral prefrontal cortex, *DN4* Douleur Neuropathique en 4 Questions, *NRS* Numerical Rating Scale, *rTMS* repetitive transcranial magnetic stimulation, *ODI* Oswestry Disability Index, *BDI* Beck’s Depression Inventory, *VAS* visual analog scale

In one of the recent studies, HF-rTMS was administered to 36 patients with chronic central neuropathic pain in motor cortex M1, with 2 randomized phases spaced 3 weeks apart. Each phase included 4 consecutive rTMS sessions and 1 final evaluation session. The final results showed a significant analgesic effect in the active phase with a 33.8% confidence interval (CI), compared to the sham phase with only 13.02% CI. And no side effect effects were observed [161, 162].

Another research showed a significant pain reduction after repeatedly applying HF-rTMS to the primary cortex M1 for 25 weeks [162]. Interestingly, the reduction in pain intensity was not apparent after the initial 5 daily rTMS sessions but was observed after 4 weeks of treatment with eight rTMS sessions. This resulted in significant pain relief over the 25 weeks of rTMS treatment. At week 25, which is 3 weeks after the last rTMS session, the percentage of patients who completed the treatment and experienced more than a 50% reduction in pain was 44.7% for M1-rTMS and 12% for sham-rTMS. These results demonstrated the clinical relevance of rTMS in treating neuropathic pain. In addition, during the first 4 weeks of the treatment, patients reported an improvement in fatigue from their diaries, indicating that rTMS may also help alleviate chronic fatigue caused by neuropathic pain.

A large number of studies were conducted on rTMS from 2013 to 2022. Based on these studies, the most serious adverse effect is seizures; other than that, hearing impairment and short-term decline in cognition were reported, while minor side effects are local pain, headache, and discomfort. However, the occurrence of side effects is rare. Most patients who received TMS treatment did not experience unpleasant side effects, which suggests that it is safe to use in treating various disorders, including chronic neuropathic pain and major depressive disorder [163].

From the numbers of the studies, it has found that anyone with any condition who undergoes rTMS delivery may be at risk of TMS-induced seizures, ranging from healthy individuals to those with neurological (e.g., post-stroke, multiple sclerosis, traumatic brain injury, meningoencephalitis, and brain tumors) or psychiatric disorders (e.g., major depression, schizophrenia, bipolar disorder, dementia, and alcohol abuse). Certain factors have been suggested to increase the chance of TMS-induced seizures. For instance, certain medications and medical conditions that lower the seizure threshold may increase the chance of seizures caused by TMS stimulation. In theory, first-degree relatives of persons with epilepsy may also have a higher possibility of TMS-related seizures, yet no such event has been observed and reported so far. Apart from the previously mentioned risk factors, sleep deprivation is considered to be of particular relevance. Some studies have reported an increase in cortical excitability, which was monitored with EEG during TMS stimulations. The same results were found in healthy individuals as well.

Although hearing loss is categorized as one of the risk factors, it is usually due to loose ear plugs when rTMS is being administered. Some individuals whose ear plugs slipped out during the procedure have experienced transient increases in auditory thresholds; therefore, hearing safety measures should be taken seriously. In terms of cognitive TMS effects, experimental studies have observed short-term cognitive decline, with degrees of decline generally considered low to moderate. However, in clinical studies, no cognitive changes were found. Despite no evidence suggesting that TMS leads to cognitive impairment. Therefore, it is recommended that further studies should be conducted to evaluate the long-term impact of TMS on cognition.

#### Cranial electrotherapy stimulation

The idea of CES was initially developed in Russia in the 1950s as a therapy for anxiety, depression, and insomnia, to be later used in pain treatment [134, 164]. The first device was called the Somniatron and first appeared in the USA in the 1970s [164]. The technique involves using a low-intensity electrical current to stimulate the cerebral cortex by application of electrodes to the patient’s earlobes (in a few reports, electrodes were attached to the mastoid processes and forehead) [134]. The current intensity is in the range of 50 μA to 4 mA [164], commonly under 2 mA (as in techniques such as tDCS and tRNS [165].

CES demonstrated satisfactory results as a treatment for depression, insomnia, post-traumatic stress disorder, and pain [164]. Moreover, the effectiveness of CES in anxiety treatment has been proven successful [165, 166]. Furthermore, this method also can be used in the pretreatment of preoperative anxiety [167].

The mechanisms underlying CES effects are currently unknown [164, 168]. CES effectiveness is suggested to be the result of the modulation of brain networks among the hypothalamus, limbic system, and reticular activating system. Moreover, imaging studies suggest that stimulation of the motor complex can result in modulating networks responsible for pain processing (thalamus), facilitating pain inhibitory mechanisms, and consequently reducing pain [134]. CES usage induces significant changes in a patient’s EEG. Alpha activity increases during stimulation, which correlates with relaxation, while beta and delta activity is decreased, which indicates a reduction in anxiety, ruminative thoughts, and fatigue [169]. Moreover, there is a supposed correlation between mechanisms of anxiety and chronic pain, having some data confirmation [165]. In this case, treatment aimed at an anxiety-causing mechanism could improve chronic pain as well. In patients experiencing chronic anxiety, enhanced chronic pain is often reported. Similarly, high preoperative anxiety levels can relate to increased pain sensing after surgery and more painful recovery. Indeed, it has been proven that anxiolytic treatments (medications and procedures) are beneficial in managing chronic pain [170]. These clinical observations were confirmed by human brain imaging studies, especially showing the role of the ACC, which is activated during anticipation of pain and influences anxiety perception (there is also a role of the amygdala and insular cortex) [170]. The mechanism that CES is believed to reduce anxiety levels may result from increased release of serotonin, endorphins, melatonin, and the concentration of γ-aminobutyric acid after stimulation [165, 169]. Therefore, this may be one of the mechanisms that CES is believed to improve chronic pain.

Another theoretical explanation for the mechanism of CES emphasizes the role of anti-nociceptive system activation, increasing serotonin, endorphins, and noradrenaline levels [171]. It has been observed that endorphin levels, decreased in the cerebrospinal fluid in patients with chronic pain, were rapidly increased after CES [171]. Moreover, the presence of endorphins in anti-nociceptive structures of the brain also confirms that hypothesis.

There exist few recent studies investigating the impact of CES on chronic pain. Older studies seemed to have proven benefits of this type of stimulation, but recent meta-analyses are not that optimistic. It has to be mentioned that there is a lack of large RCTs that could verify CES use in chronic pain treatment. Among available trials (Table 5), there is often a high risk of bias, and therefore the quality of evidence is low, which was emphasized by mentioned meta-analyses.

**Table 5:** Summary of clinical trials from the last 10 years (2013–2022) investigating cranial electrotherapy stimulation (CES) and reduced impedance non-invasive cortical electrostimulation (RINCE) for various chronic pain conditions

Study	Country	Stimulation technique	Study design	Sample size	Chronic pain condition	Main results	Adverse events
Ahn 2020 [172]	USA	CES	RCT	30	Knee osteoarthritis	Most measurements of clinical pain severity and experimental pain sensitivity differed significantly between the active and sham groups (Cohen’s *d* = 1.43, *p* < 0.01 for NRS; *d* = 1.40, *p* < 0.01 for heat pain threshold; *d* = 1.50, *p* < 0.01 for pressure pain threshold; and *d* = 1.95, *p* < 0.01 for conditioned pain modulation); NRS change: sham 5.73 ± 16.02 vs active group −17.00 ± 15.79. CES reduced clinical pain severity and increased heat pain threshold, PPT, and CPM.	N/A
Yennurajalingam 2018 [168]	USA	CES	Pre-post intervention study	33	Advanced cancer	The number of patients who achieved 25% and 50% decrease, respectively, in symptom intensity and distress after CES treatment were as follows: depression (HADS) 56%, 53%; anxiety (HADS) 56%, 28%; sleep quality (PSQI) 41%, 21%; pain severity (BPI) 52%, 21%; distress scores (distress thermometer) 53%, 35%.	N/A
Taylor 2013 [173]	USA	CES	RCT	46	Fibromyalgia	Individuals using the active CES device had a greater decrease in average pain (*p* = .023), fatigue (*p* = .071), and sleep disturbance (*p* = .001) than individuals using the sham device or those receiving usual care alone over time.	N/A
Hargrove 2012 [174]	USA	RINCE	RCT	77	Fibromyalgia	Mean tender points in the AT group dropped significantly, representing an average improvement of 43%. In contrast, no change was found for the PL group. 59.0% of subjects in the AT group achieved pressure pain threshold improvement of at least 50%, while only 2.6% of those in the placebo group experienced such change. Pain VAS score (0–10): −2.0 in AT vs −0.6 in PL (*p*=0.03).	Short-lived headache (*n*=2), eye movement/flutter during treatment (*n*=1), increased perception of restlessness (*n*=1), nausea (*n*=1)

*RCT* randomized controlled trial, *CES* cranial electrotherapy stimulation, *RINCE* reduced impedance non-invasive cortical electrostimulation, *NRS* numerical rating scale, *VAS* visual analog scale

Besides some RCTs included in this paper, there are also systematic reviews worth mentioning, as the number of adequate RCTs is low and seems to need completing. In a systematic review by Shekelle, according to the analysis, there were again a small number of trials, and the risk of bias was high. The results evaluating the effectiveness of CES in chronic pain conditions were diverging. Some trials taken into analysis showed no statistically significant difference in pain scores compared to sham groups. Contrarily, other studies reported improvement in patients with such conditions as fibromyalgia, chronic neuromuscular pain, and musculoskeletal pain. Due to the high risk of bias, current evidence for CES effectiveness is considered insufficient [175]. Another study by this author presented similar conclusions [176].

Another systematic review found no improvement in chronic pain after using CES. This study aimed to determine the benefits of non-invasive brain stimulation methods in neuropathic pain after spinal cord injury. However, CES did not demonstrate any effects on pain and depression in examined patients [165].

A randomized controlled pilot study from 2020 examining the efficacy of CES in adults with osteoarthritis showed a positive impact on decreasing chronic pain. Active CES contributed to the reduction of scores on the Numeric Rating Scale. This randomized, double-blind, sham-controlled clinical trial featured remotely supervised CES, for 60 min daily, over 2 weeks (Monday to Friday), after previous proper instruction. The CES electrodes were attached to the patient’s earlobes. No adverse effects were reported during the trial. This study also figured out the difference in the functionality of the frontal cortex during active CES versus sham and under pain stimuli. This could suggest another mechanism of CES in reducing pain, as it induces a decrease in oxygenated hemoglobin in the frontal cortex. After all, CES in this study has been proven effective in managing chronic pain in OA. Moreover, it can be used remotely [172].

A study aiming to investigate the effects of CES therapy on symptoms in fibromyalgia showed a higher decrease in pain, fatigue, and sleep disturbance in the active CES treatment group, compared to the patients receiving either sham or usual care [173]. Therefore, the functional status of the active CES device group has increased. The study also investigated the effects of CES on both systolic and diastolic blood pressure, demonstrating no influence on these parameters and thus showing the safety of such therapy.

CES may also be beneficial for patients with advanced cancer, according to the results of a preliminary study [168]. The study has found that using CES in advanced cancer is safe for the patients and improves pain associated with the disease. After 4 weeks of treatment, there was a significant improvement in pain severity. Although these findings may seem promising, there were limitations in the study. The important one was the lack of a sham group, resulting in low reliability and a need for further well-designed research.

According to the current evidence, there have been no serious adverse events after CES using treatment. Adverse events such as pulsing, tingling, and tickling in the ears; tender ears; pins; and needles feeling near the bladder were reported in the group of actively stimulated patients versus drowsiness, warm ears, and headache after one session in the sham group (rare events, only in one participant each) in one study [177]. Also, one other study included information about adverse events, though these were mild such as sensations of ear pulsing, stinging, itching, electric sensations, or ear clip tightness [134, 178]. In general, these are the main adverse events we can expect.

In one early study, some patients experienced worsening depression after active CES, but it was probably related to the old type of used devices, no longer in use [179]. Other observed adverse effects were tiredness, malaise, sleepiness, skin irritation, and possible transient visual symptoms [176, 180], although another study revealed no reported adverse events during CES treatment [171].

#### Reduced impedance non-invasive cortical electrostimulation

RINCE is a much less described method of electrostimulation than previous ones. The current in this technique is applied by electrodes attached to the patient’s scalp. The main difference from the other stimulation techniques is that it uses specific current frequencies that allow deeper cortical penetration by reducing the impedance of the skin and skull. This enables low-frequency cortical modulation and is believed to increase signal transmission and stimulation effectiveness [134].

The effectiveness of RINCE in reducing pain was mentioned in the meta-analysis by O’Connell et al. It included only two articles regarding RINCE in chronic pain due to the lack of existing literature and studies (Table 5). In one of these studies, primarily unpublished (Deering 2017), the short-term follow-up demonstrated a positive impact on pain intensity. However, the rank of proof was poor. An improvement in quality of life was not observed [134]. Moreover, none of the included studies have shown any superiority of stimulation over sham. The study that we could reach [174] was a RCT investigating RINCE in managing symptoms of fibromyalgia patients. The results suggest a positive impact of active RINCE therapy on managing fibromyalgia symptoms. During treatment, both mean tender points and pressure pain threshold represented an improvement in the active group compared to the sham group. There was also a decrease in pain VAS scores in patients receiving active RINCE treatment. The outcomes were statistically and clinically significant, suggesting the advantages of using a RINCE device in such conditions.

Adverse events after RINCE include headache (mild to moderate intensity), nausea, dizziness, vertigo, and localized skin reactions. These side effects were mild to moderate and occurred averagely with a frequency of two complications per patient, but fortunately were short-lived and disappeared with no intervention. Other events reported by another included study were eye movement and restlessness, with low incidence [134].

## Conclusion

In recent decades, numerous researchers extensively debated the clinical usefulness of brain stimulation as a treatment for chronic pain.

The exact phenomena underlying the analgesic mechanisms of brain stimulation are still unrecognized in the case of all techniques discussed in this review. However, various hypotheses on their probable mechanism of action have been formed in recent decades, and evidence in this field constantly grows. The enhancement of knowledge about pain relief mechanisms by brain stimulation may significantly contribute to improving the current clinical outcomes of this approach.

The majority of studies conducted to date on the use of brain stimulation in chronic pain are burdened with small sample sizes and poor scientific methodology.

DBS stands out from the rest of the methods in its efficacy in the treatment of cluster headaches. Regarding MCS, there is inconsistency as regards the results of published to-date clinical studies. The identification of specific subgroups of patients well responding to MCS is still under investigation. Although due to the high complication rate of invasive brain stimulation methods, their use should be appropriate only when the chronic pain is refractory to other available therapeutic methods.

Since tDCS and rTMS demonstrated successful results on chronic pain relief with a low rate of side effects, their clinical use is the most beneficial among discussed techniques. Moreover, current research on adverse effects prevention is continued to further increase the safety of these methods.

Regarding CES and RINCE, it is clear that there is a need for further research to confirm findings and their reliability because the quality of evidence remains very low. There are only a few studies that suggest CES therapy to be beneficial and most clinical trials showed no benefit. RINCE was proven effective in managing chronic pain, but data referred only to one study, so there was the risk of bias due to the small sample size.

In summary, rTMS and tDCS represent the most promising therapeutic options for chronic pain among discussed brain stimulation methods. However, due to the low quality of evidence provided by available studies, large multi-center RCTs with long-term follow-ups are necessary to verify the safety and clinical outcomes of non-invasive as well as invasive brain stimulation.

### Supplementary Information

ESM 1: Flowchart: Flow diagram of the literature search strategy (DOCX 28 KB)

#### CRediT author statement

**Michał Szymoniuk:** conceptualization; methodology; investigation; data curation; writing—original draft preparation; writing—review and editing; **Jia-Hsuan Chin:** investigation; data curation; writing—original draft preparation; visualization; **Łukasz Domagalski:** investigation, data curation, writing—original draft preparation; **Mateusz Biszewski:** investigation, data curation, writing—original draft preparation; **Katarzyna Jóźwik**: investigation, data curation, writing—original draft preparation; **Piotr Kamieniak:** writing—review and editing, supervision, project administration. All authors have read and agreed to the published version of the manuscript.
