# Supplementary material for: Brain stimulation for chronic pain management: a narrative review of analgesic mechanisms and clinical evidence
Source: Neurosurg Rev. 2023 May 29;46(1):127. doi: 10.1007/s10143-023-02032-1 (PMC10227133; doi:10.1007/s10143-023-02032-1)
Supplement: Supplementary file 1 [file 10143_2023_2032_MOESM1_ESM.docx]

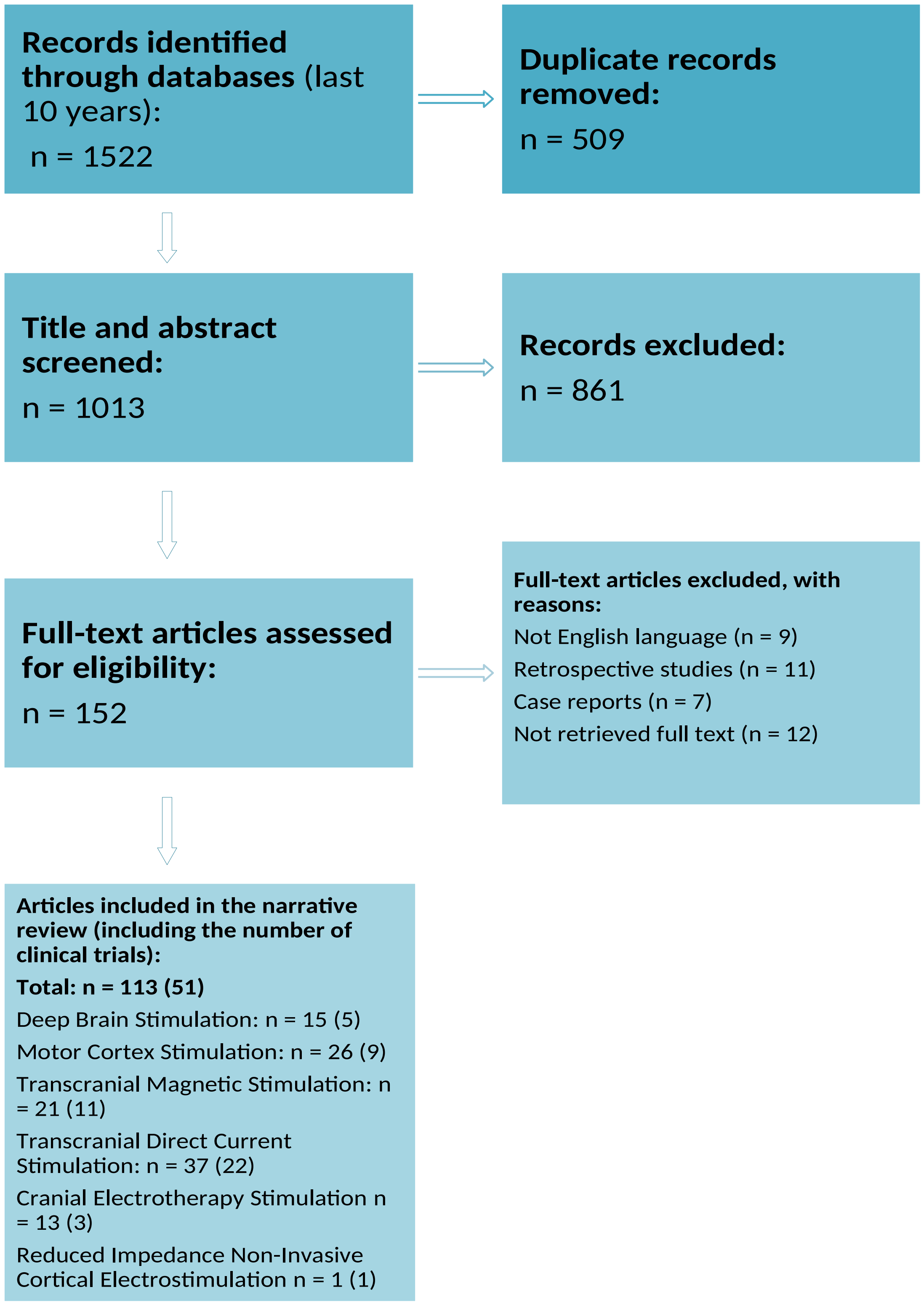
Records identified through databases (last 10 years):
n = 1522
Duplicate records removed:
n = 509
Title and abstract screened:
n = 1013
Records excluded:
n = 861
Full-text articles assessed for eligibility:
n = 152
Full-text articles excluded, with reasons:
Not English language (n = 9)
Retrospective studies (n = 11)
Case reports (n = 7)
Not retrieved full text (n = 12)
Articles included in the narrative review (including the number of clinical trials):
Total: n = 113 (51)
Deep Brain Stimulation: n = 15 (5)
Motor Cortex Stimulation: n = 26 (9)
Transcranial Magnetic Stimulation: n = 21 (11)
Transcranial Direct Current Stimulation: n = 37 (22)
Cranial Electrotherapy Stimulation n = 13 (3)
Reduced Impedance Non-Invasive Cortical Electrostimulation n = 1 (1)
